# Fabrication of Low Roughness Gradient Nanostructured Inner Surface on an AISI 304 Stainless Steel Pipe via Ultra-Sonic Rolling Treatment (USRT)

**DOI:** 10.3390/nano11071769

**Published:** 2021-07-07

**Authors:** Xiaolei Han, Changji Li, Chunhuan Chen, Xiaodan Zhang, Hongwang Zhang

**Affiliations:** 1 National Engineering Research Center for Equipment and Technology of Cold Strip Rolling, College of Mechanical Engineering, Yanshan University, Qinhuangdao 066004, China; m18833531804@163.com; 2 Shenyang National Laboratory for Materials Science, Institute of Metal Research, Chinese Academy of Sciences, Shenyang 110016, China; cjli@imr.ac.cn; 3 School of Materials Science & Engineering, Dalian Jiaotong University, Dalian 116028, China; chchen@djtu.edu.cn; 4 Department of Mechanical Engineering, Technical University of Denmark, 2800 Kongens Lyngby, Denmark; xzha@mek.dtu.dk

**Keywords:** gradient nanostructure, AISI 304 stainless steel, ultra-sonic rolling treatment, surface quality

## Abstract

Gradient nanostructure (GNS) has drawn great attention, owing to the unique deformation and properties that are superior to nanostructure with uniform scale. GNS is commonly fabricated via surface plastic deformation with small tips (of balls or shots) so as to produce high deformation to refine the coarse grains, but unfortunately it suffers from the deterioration of surface quality which is hard to guarantee the reliable service. Although there are mirror-finishing techniques that can greatly enhance the surface quality, the induced slight deformation is commonly unable to produce GNS of reasonable thickness. Here, we propose a method to fabricate a GNS surface layer with a substantially enhanced surface quality via ultra-sonic rolling treatment (USRT), namely, surface rolling with a roller vibrated at a frequency of 20,000 Hz. It is found that 4-pass USRT is able to produce 20–30 µm thick GNS on AISI 304 stainless steel pipe inner surface, wherein the surface quality is enhanced by one order of magnitude from the starting *Ra* = 3.92 µm to 0.19 µm. Processing by a roller with a high-frequency vibration is necessary for both good surface quality and the effective accumulation of heavy deformation on the surface. The flattening mechanism as well as the microstructural evolution from millimeter- to nanometer-scale for AISI 304 stainless steel is discussed.

## 1. Introduction

GNS, i.e., a nanostructure with continuously varied characteristic size within certain length scale, exhibits deformation behaviors and mechanical properties that are different from but usually superior to those of nanostructures with relatively uniform size distribution [1,2]. In the past decades, various techniques have been developed to fabricate GNS, among which surface gradient plastic deformation has drawn great attention due to its wide applicability, high efficiency, low cost, and more importantly its lack of introducing artificial defects and contaminations. Deformation-induced nanostructuring lies in the refinement of the coarse-grains down to nanometer-scale via applying severe plastic deformation, and the existing techniques have thus been developed with the aim to produce deformation as heavy as possible. For instance, the most representative methods to fabricate GNS, such as surface mechanical attrition treatment (SMAT) [3] and surface mechanical grinding treatment (SMGT) [4], have been designed through penetrating and/or shearing the sample surface with ball tips. High stress generated due to small contact area enables the activating high density of crystal defects to refine the coarse grains down to nanometer regime. However, it is disadvantageous to the surface quality, which is very important for higher service reliability and longer service life.

Mirror surface with small roughness can be produced by surface polishing or grinding with a mechanical [5], chemical or electropolishing [6], magnetic [7], or laser [8] manner, and by surface rolling [9]. The polishing or grinding enhances surface quality through removing the convex peaks and producing debris via cutting or plastic deformation by using polishing materials such as whetstone, wool wheel, or sandpaper. On the contrary, the surface rolling reduces roughness by flattening asperity peaks via cold deformation induced by high pressure, namely, a debris-free process. A smooth surface with uniform small roughness requires large contact area by which large surface plastic deformation can be avoided as much as possible. This is apparently not suitable for producing thick GNS that needs heavy plastic deformation. Consequently, GNS with good surface quality is traditionally unreachable at the same time.

Actually, large plastic deformation can also be generated in case small deformation is highly accumulated, and potentially be able to produce GNS with good surface quality. Such a purpose may be realized by cyclic loading with ultrahigh frequency, during which small deformation induced by each cycle may be fit for good surface quality, while quickly accumulated large deformation should be good for microstructural refinement. Ultrasonic processing is such a technique that deforms sample surface with shots, needles, pins, or rods, tens of thousands of times per second, which has drawn extensive interest in the past century in many industrial applications such as ship, marine, and automotive [10]. Recently, ultrasonic processing has been developed, such as ultrasonic peening treatment (UPT), ultrasonic shot peening (USSP), and ultrasonic cold forging technology (UCFT) [10,11,12], to produce nanostructured surface layer in engineering components so as to improve their mechanical properties. Ultrasonic processing has been reported to substantially improve the surface quality compared with the traditional mechanical polishing process [13]. In the present study, we demonstrated that an ultrahigh frequency surface processing with line-contact roller ultrasonic rolling treatment (USRT) is able to fabricate GNS with reasonable thickness and good surface quality. The mechanisms responsible for the surface flattening and nanostructuring are addressed.

## 2. Materials and Methods

**Figure 1:**
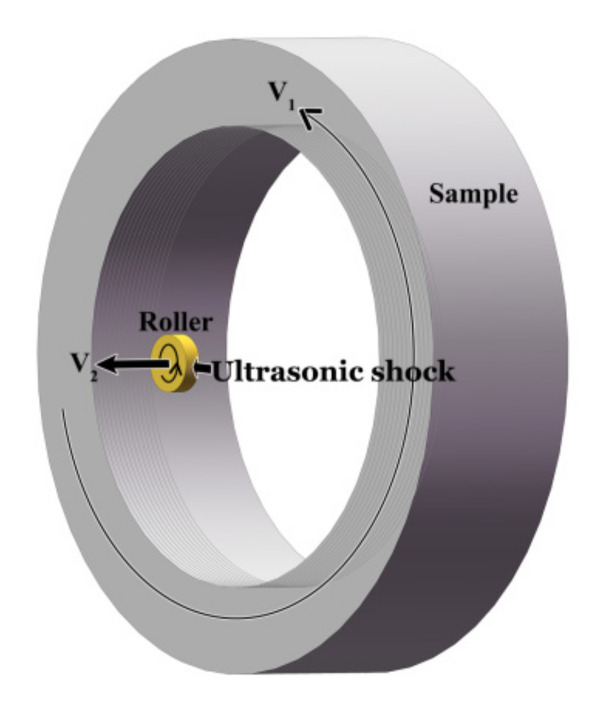
Schematic illustration of the USRT set-up.

An AISI 304 stainless steel pipe with 80 mm in inner diameter and 108 mm in outer diameter was chosen as the experimental material. The chemical composition (in wt.%) is as follows: 0.049 C, 18.46 Cr, 8.28 Ni, 0.012 Mo, 1.64 Mn, 0.42 Si, 0.021 P, 0.03 S, and balance Fe. The USRT was performed with H + C6136A/30E Huawin Hawking. The starting material was firstly subjected to solution treatment, namely, 1080 °C for 1 h, followed by water quenching to produce a single-phase face centered cubic (fcc) austenite with an average grain size of 50 µm. USRT was designed to roll the sample surface with a roller (the surface roughness *Ra* ≤ 0.15 µm) that simultaneously vibrates at an ultrahigh frequency. As shown by Figure 1, the roller of 30 mm in diameter and 2 mm in width was connected with an ultrasonic generator that produces a vibration of 20 kHz. After the roller was closely contacted with the sample surface, extra penetration was added to generate a loading on the sample surface. The roller rotates with the pipe at a speed of *V*_1_ and moved simultaneously along the pipe axis at a speed of *V*_2_. The sample surface was thus treated from one end to the other, undergoing 1-pass processing. Here, in the present investigation, the pipe inner surface was subjected to 4-pass processing with *V*_1_ = 68 rpm, *V*_2_ = 10 mm/min, and a penetration depth of 80 µm.

Microstructural characterization was performed on the cross section by a FEI-Scios scanning electron microscope (SEM) and a FEI-Talos transmission electron microscope (TEM) operated at 200 kV(Thermo Fisher Scientific, Waltham, MA, USA). Cross-sectional samples were prepared through the following procedures: (i) electro-deposition of protecting Ni layer onto the treated surface; (ii) cutting 0.8 mm thick slices and mechanical thinning down to 40 µm; (iii) final thinning by the double-jet electrolytic polishing (electrolyte: 10 vol.% HClO_4_ + 90 vol.% C_2_H_5_OH, 0 °C) supplemented with the ion-milling (Leica 102) to generate large transparent areas. Surface roughness was determined by a TIME 2000 surface roughness tester (JITAI KEYI, Beijing, China) with a resolution of 0.01 µm, where the sampling length was 0.8 mm and the roughness parameters were evaluated 5 times the sampling length, i.e., 4 mm. The hardness was measured by a Qness Q10A+ microhardness tester (Qness GmbH, Golling, Austria) with a loading of 0.25 N and a holding time of 10 s.

## 3. Results

### 3.1. Surface Quality 

**Figure 2:**
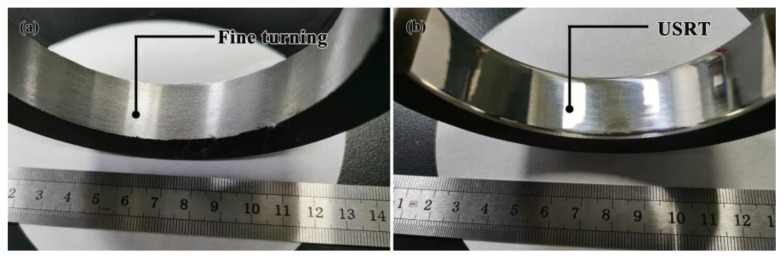
Comparison of the surface quality of AISI 304 stainless steel before (**a**) and after (**b**) the USRT processing.

From Figure 2, the surface quality has been greatly enhanced after USRT processing since a mirror surface has formed. Quantitatively, the surface roughness was evaluated according to the arithmetic mean height from the base line, *Ra*; the distance between the highest peak and the lowest valley, *Rz*; the sum of the mean for 5 maximum height and that for 5 minimum height, *Rz* (JIS); the average peak distance, *Rs*; and the maximum peak height, *Rp*. As shown by Table 1, *Ra*, *Rz*, *Rz* (JIS), and *Rp* decreased by a factor of 13.4 to 23.3, while *Rs* decreased by a factor of 1.4, pointing to the substantially decreased the height of roughness by USRT. Comparatively, rolling treat (RT) without ultra-sonic vibration slightly changed the surface quality, wherein *Ra* was 7.8 times larger than that for USRT.

### 3.2. Cross-Sectional Microstructure and Hardness

**Figure 3:**
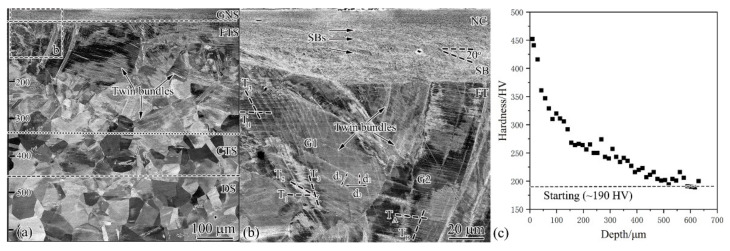
Cross-sectional SEM observations (**a**), the enlarged image of the rectangle area (**b**), and the variation of Vickers hardness as a function of depth (**c**) for the USRT AISI 304 stainless steel. Note that GNS was followed by SB (shear bands), FTS (fine-twinned structure), CTS (coarse-twinned structure), and DS (dislocation structure). Hardened layer of 600 µm thick was produced, with the maximum of 450 HV.

The microstructure was characterized from the cross-section perpendicular to the pipe axis. As shown in Figure 3a, 600 µm thick deformation layer was observed and composed of three distinct regions: gradient nanostructure (GNS), twinned structure (TS), and dislocation structure (DS). GNS was formed on the topmost surface of 20–30 µm thick with uniform contrast. From the enlarged image (Figure 3b), one can see that GNS was actually composed of nanocrystalline (NC) and shear bands, but unfortunately their structural details were unable to be addressed by SEM (see the latter TEM characterization). TS covered a large area from 30 to 450 µm, with single or multiple deformation twins formed in each austenitic grain. TS area can be further divided into fine-twinned structure (FTS) and coarse-twinned structure (CTS) according to the twin thickness. CTS was formed in the deeper layer mainly with evenly distributed thick twins, whereas FTS was at smaller depth, characterized by thin twins. Twins for some grains in FTS were unevenly distributed and presented in the form of twin bundles, as demonstrated by G1 in Figure 3b with twin bundle that was actually composed of three or more thin twins. The twin spacing d_1_, d_2_, and d_3_ decreased with the approach to the surface. Twins can also be evenly distributed, as shown by G2 grain with single twins. Close observation found that T_A_ showed increasingly finer spacing with a decrease of depth, whereas T_B_ changed slightly. DS was induced beyond a thickness of 450 µm, which was characterized by high density of dislocations, giving rise to contrast difference within grin interiors.

From Figure 3c, one can see that the top surface of 20–30 µm, namely, GNS, showed a hardness of 370–450 HV that is about 2 times the value (190 HV) of the original coarse-grained counterpart. In the depth range of 30–450 µm, namely, TS, the hardness drops continuously to 220 HV. Very slight hardening is observed in the depth range of 450–600 μm, corresponding to the DS area with a hardness of 190–220 HV. The present hardening was significant compared to AISI 304 stainless steel subjected to the high-speed shearing with ball tip, namely, the pipe-inner surface grinding (PISG, 1-pass *V*_1_ = 80 rpm, *V*_2_ = 50 mm/min, and a penetration depth of 40 µm), by which a highest hardness of 400 HV and 400 µm hardened layer were produced [14]. It is also more effective than that for AISI 304 stainless steel processed by high-speed shot blasting via surface mechanical attrition treatment for 15 min, wherein the maximum hardness was 320 HV and a hardened layer was 450 µm thick [15]. Here, we underpin the fact that the USRT-produced GNS can simultaneously enhance surface quality and hardening.

### 3.3. XRD Examination

**Figure 4:**
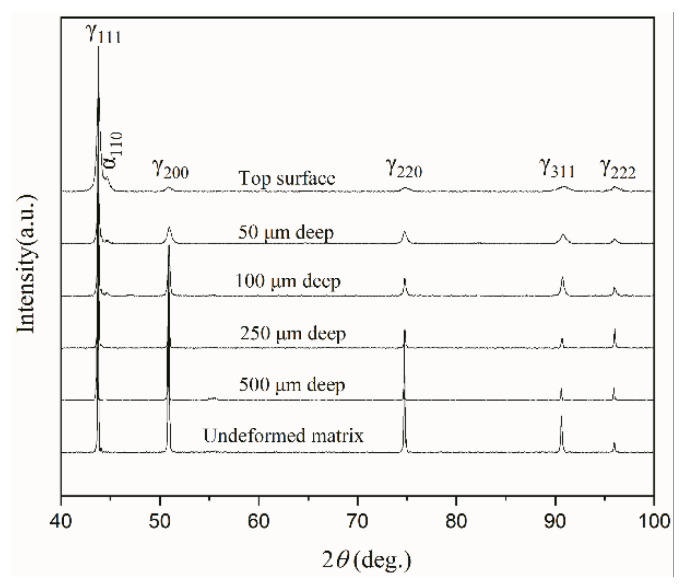
XRD profiles of the AISI 304 stainless steel sample before and after USRT. The profiles for different depths were obtained by removing surface layer of corresponding thickness. γ refers to fcc austenite, while α refers to the bcc martensite.

Figure 4 shows that the starting AISI 304 stainless steel is a single-phase fcc austenite (γ). After USRT, all the diffraction peaks are broadened, and the broadening becomes more and more pronounced with the decrease of depth. This implies that the accumulation of crystal defects and the structural refinement became more and more significant when approaching the sample surface. Moreover, an extra diffraction peak occurs and can be indexed to be the body-centered cubic (bcc) phase, namely, deformation-induced martensite that is well documented during the plastic deformation of metastable austenitic steel [16]. Comparatively, here, the martensitic transformation was less significant, since only the strongest (110) peak has been detected with a very low intensity, while the rest bcc peaks are all absent. With an increase of depth, (110) peak became more and more weak until it disappeared completely at a depth >250 µm.

### 3.4. TEM Characterization

**Figure 5:**
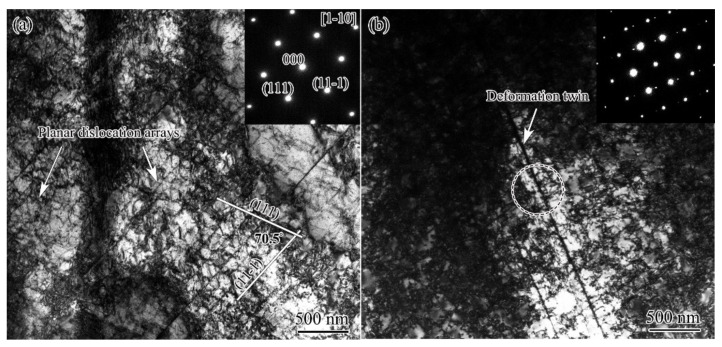
TEM observation of the microstructure of DS (**a**) and CTS (**b**) of the USRT AISI 304 stainless steel sample.

DS for AISI 304 stainless steel is the typical planar dislocation array, namely, dislocations arrange themselves in the planar configuration on their respective primary slip planes. As seen from Figure 5a, two sets of planar dislocation arrays were observed intersecting by an angle of 70.5°, corresponding to the angle between (111) and (11-1) slip planes. A deformation twin in the CTS region was shown by Figure 5b, which produced the weak diffraction patterns in the inserted selected diffraction pattern (SAED). The weak and strong diffraction patterns were mirror symmetric with respect to the twin plane normal. It was the typical feature of the electron diffraction for fcc twin and matrix [17]. Here, the twin density was very low, and the matrix contained a high density of planar dislocation arrays.

**Figure 6:**
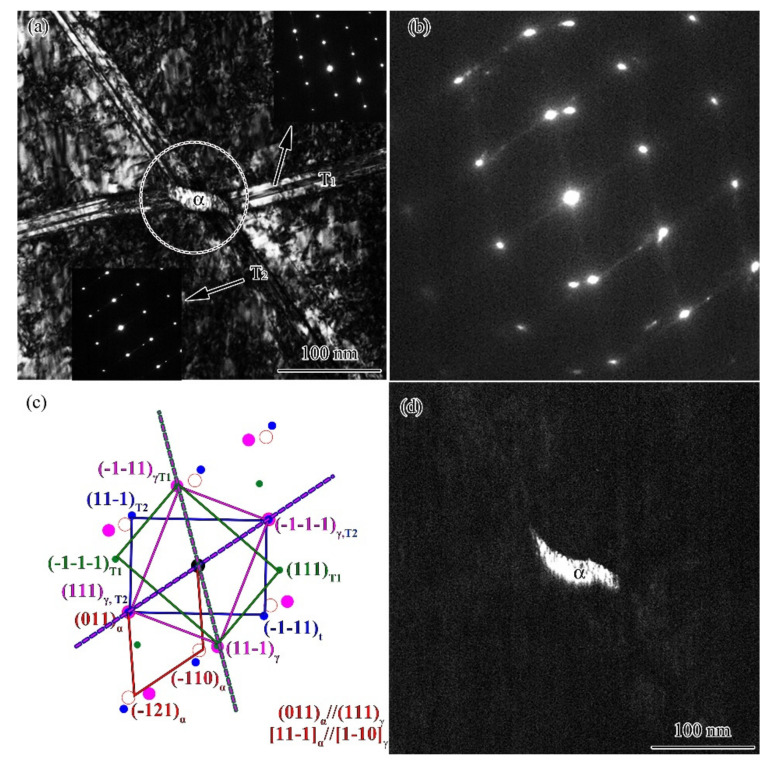
TEM bright filed image (**a**), SAED pattern (**b**), the indexed sketch map (**c**), and the dark field image of martensite (**d**) USRT AISI 304 stainless steel sample (α refers to the bcc martensite).

The twin–twin intersection is very common for TS when multiple twins form, and deformation-induced martensitic transformation usually occurs at the intersection point [15,16]. As shown in Figure 6a and the inserted SAED patterns, two sets of twins (T_1_ and T_2_) intersect. The SAED patterns (Figure 6b,c) from the circled area was overlapped by three patterns, namely, T_1_-matrix, T_2_-matrix, and bcc [11-1]. The dark field image, Figure 6d, is obtained using the diffraction spot of bcc (−110)_α_, pointing to the martensitic transformation at the intersection point. From the SAED pattern, martensite and austenite held the orientation relationship: (011)_α_//(111)_γ_ and [11-1]_α_//[1-10]_γ_, which was known as the Kurdjumov–Sachs (K-S) orientation relationship [18]: {011} _α_//{111}_γ_, <111 > _α_//<011>_γ_. Such an orientation relationship was also observed in the PISG processed AISI 304 stainless steel sample [14].

**Figure 7:**
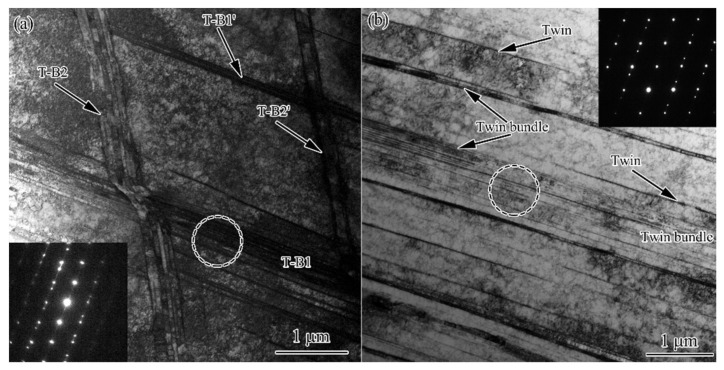
TEM observation of the intersection (**a**) and single (**b**) twin bundles for FTS in USRT AISI 304 stainless steel sample. Circles indicate the areas where the SAED patterns were obtained.

The twin bundle is a typical feature for the FTS. TEM observations show that twin bundles may contain several to tens of twins with very small thickness. Figure 7a shows the intersection of two sets of twin bundles (T-B1 and T-B2) within an area around 20 µm^2^. It was interesting to find that the twin bundles at the top-right corner (T-B1’ and T-B2’) each contains two twins, whereas those at the bottom-left corner (T-B1 and T-B2) have several to 20 of them. The continuous formation of more twins close to the original ones is thus underpinned, since no twins are observed in the areas between the parallel bundles. Such multiplication can be more clearly observed in Figure 7b, where one set of twins is developed. Note that new twins form around the original one so as to form a twin bundle, while single twins remain in the adjacent region. Through the continuous formation of new twins, the structural scale is substantially reduced.

**Figure 8:**
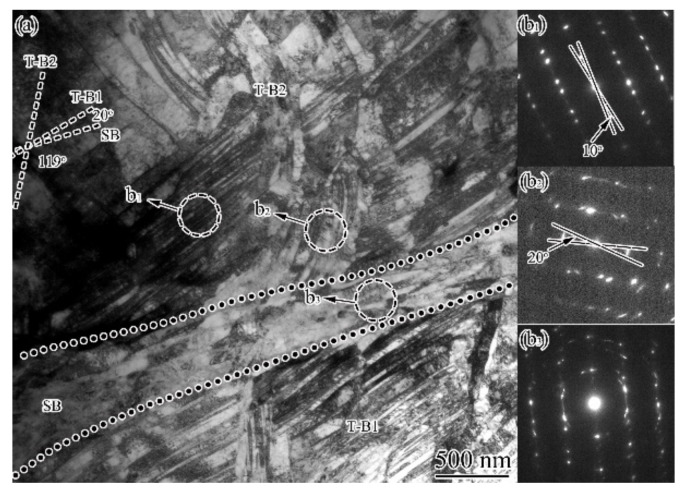
TEM observation (**a**) and the corresponding SAED patterns (**b_1_**–**b_3_**) for the SBs within FTS in USRT AISI 304 stainless steel sample. Circles indicate the areas where the SAED patterns were obtained.

When approaching the sample surface, twin bundles were subjected to further deformation and shear bands developed. From Figure 8a, one can see that twin bundles exhibited orientation change of 10–20°, as reflected by the SAED patterns (Figure 8b_1_,b_2_), where the diffraction spots for both twin and matrix were elongated and deviated the standard twin-matrix relationship. The twin bundles were cut by a SB with a thickness of 400–500 nm, within which extended boundaries with the spacing of 100–200 nm were observed parallel with the SB. The SAED pattern (Figure 8b_3_) showed the coexistence of twins and dislocation boundaries with large misorientation. The trace of those extended boundaries were 20° and 61°, respectively inclined to that of the T-B1 and T-B2, which together with the apparently coarser structural scale implied that the twins were unlikely to belong to any of the outside twin bundles.

**Figure 9:**
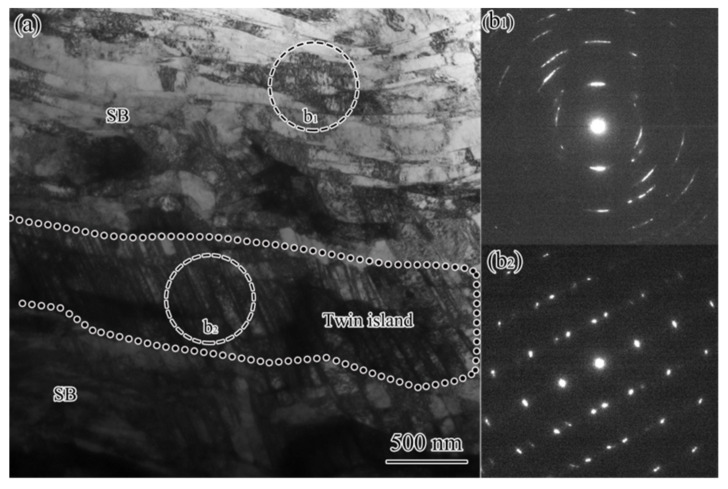
TEM observation (**a**) and the corresponding SAED patterns from SB (**b_1_**) and twin island (**b_2_**) in USRT AISI 304 stainless steel sample.

At a much smaller depth, shear bands were well developed, and the TS was consumed efficiently. As demonstrated by Figure 9, significant shearing destroyed most of the TS and left a twin island among the extended structure that was formed within SBs. The extended boundaries had the spacing spanning from tens to hundreds of nanometers, among which both high- and low-angle boundaries were presented as indicated by the discontinuous diffraction circles in the SAED pattern (Figure 9b_1_). Inside the twin island, the twin and matrix remained the twinning relationship but were subjected to slight orientation spread according to the mirror symmetric diffraction pattern with slight elongated spots, as shown in Figure 9b_2_.

**Figure 10:**
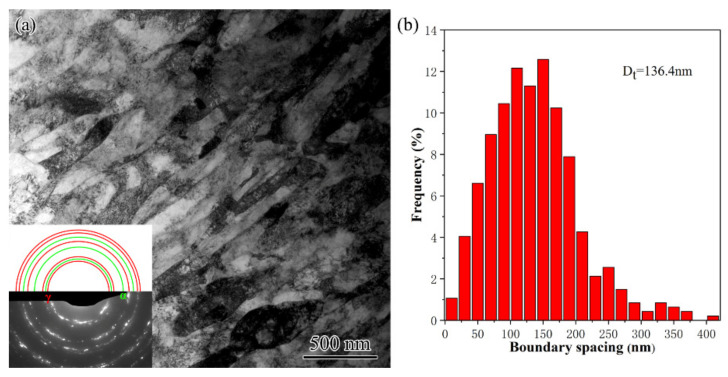
TEM observation (**a**) and the corresponding histogram of boundary spacing (**b**) of the ultrafine lamellae in USRT AISI 304 stainless steel sample. The SAED pattern together with theoretic circles for fcc γ and bcc α was inserted.

TS disappeared completely at 50 μm deep and was replaced by an extended structure (Figure 10a). The extended structure was characterized by sharp extended boundaries that were roughly parallel with the surface. According to the relatively continuous diffraction circles in the inserted SAED pattern, these extended boundaries were mainly high-angle ones. The comparison with theoretic diffraction circles for fcc γ and bcc α evidenced the presence mainly of austenite, while martensite was very insignificant. As seen in the histogram shown in Figure 10b, these extended boundaries had the spacing distributed from 20 to 400 nm with an average of 136.4 nm. The extended structure was the typical ultrafine lamellar structure (UFL) that is commonly formed in medium-to-high stacking fault energy metals subjected to high-strain deformation [4,19]. Such UFL structure was also reported in PISG-processed AISI 304 stainless steel [20].

**Figure 11:**
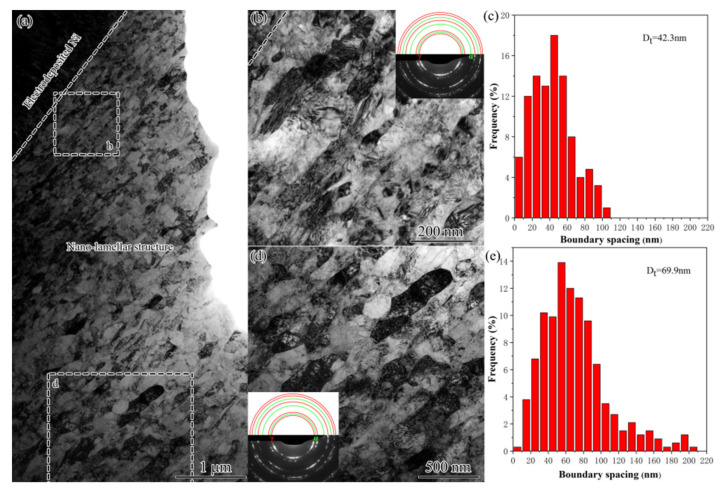
TEM observations (**a**,**b**,**d**) and the corresponding histograms of boundary spacing (**c**,**e**) of the GNS in USRT AISI 304 stainless steel sample. (**b**,**d**) The enlarged image of the rectangle marked area in (**a**). The SAED pattern together with theoretic circles for fcc γ and bcc α was inserted.

At the topmost surface of several microns thick, nanolamellar structure (NL) was observed. As demonstrated by Figure 11a,b,d, NL showed identical features as those for UFL, namely, with extended boundaries parallel with the treated surface, containing a high density of dislocations within lamellae interior, roughly random orientation with mainly high angle boundaries, dominated by fcc austenite and a very small amount of bcc martensite, but also showed the boundary spacing in the nanometer regime. The NL at the smaller depth of 1–2 µm had the boundary spacing from 5 to 100 nm with an average of 42.3 nm (Figure 11c), while for that at the relatively deeper layer of 4–5 µm spanned from 5 to 200 nm with an average of 69.9 nm (Figure 11e).

## 4. Discussion

The above experimental results demonstrate the achievement of both high surface quality and the structural refinement down to a nanometer regime by applying USRT. The unique advantage for the present processing in smoothing asperities as well as the induced evolution of microstructure from millimeter- to nanometer-scale is discussed in the following section.

### 4.1. Asperity Flattening

After USRT, the surface quality was greatly enhanced. The maximum peak height (*Rp*) decreased from 9.08 to 0.39 µm, and the distance between the highest peak and the lowest valley (*Rz*) decreased from 19.14 to 1.43 µm. Comparatively, it was more significant compared to the surface rolling with same parameters but absent of ultrasonic vibration, as both *Rp* and *Rz* were reduced by factors of 4.8 and 5.7, respectively. This points to the fact that the rolling with ultrasonic vibration is able to flatten the surface asperity more effectively. The detailed flattening process can refer to the traditional rolling process, during which three cases may exist depending upon the lubricant features [21]: (i) very thin lubricant films, the roughness of rolled strip was finally conformed to that of the roller; (ii) intermediate thick film, the strip roughness was directly related to (a quarter of) the lubricant film thickness; and (iii) thick lubricant films, the roughening resembles that of the free surface, owing to the local different deformation. The formation of lubricant films is unlikely for the present USRT, and the roughening can be assumed as case (i), namely, the flattening of the existing asperity peaks. According to the asperity hardness model proposed by Wilson and Sheu [22] to appraise the asperity’s ability to dispute the approach of tool surface,

*H* = Δ*P*/*k* = 2/[*f*_1_(*A*)*E*+*f*_2_(*A*)] (1)

where *H* is the non-dimensional asperity hardness, Δ*P* is the difference between the pressure on the asperity peak and that in the asperity valley, *k* is the bulk shear strength, *f*_1_ and *f*_2_ are functions of *A*– the contact area ratio (the real contact area to the global contact area), and *E* is the non-dimensional strain rate [22]:

*f*_1_ = 0.515+0.345*A*−0.860*A*^2^ (2)

*f*_2_ = 1/[2.571−*A*−*A*ln(1−*A*)] (3)

**Figure 12:**
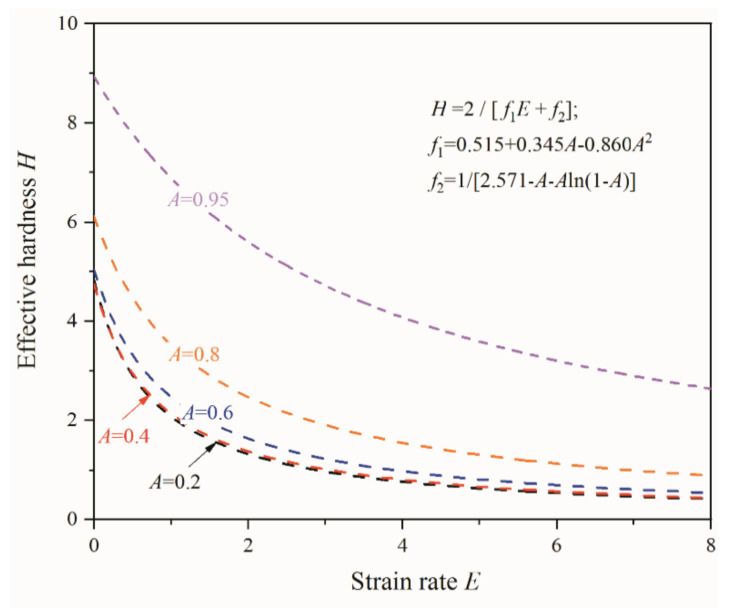
Variation of non-dimensional effective hardness (*H*) as a function of non-dimensional strain rate (*E*) given contact area ratios of 95%, 80%, 60%, 40%, and 20%.

As seen in Figure 12, the effective hardness *H* decreased monotonically with an increase of strain rate *E*, irrespective of contact area ratio, implying that surface flattening becomes easier for processing with higher strain rate. Note that the strain rate was defined as the ratio of the local bulk strain rate in the underlying materials to a typical local strain rate associated with asperity flattening or surface indentation [22]. Given local bulk strain of $\dot{\varepsilon }$, when asperities spaced by *S_m_* were flatted at a velocity of *v_fs_*, the non-dimensional strain rate *E* is

(4) $$E=\dot{\varepsilon }S_{m}/(2\;v_{\mathrm{fs}})$$

For the present inner surface rolling, the deformation was localized within the topmost thin layer, of which the bulk strain rate can be approximated according to the roughness parameters. The roughness change in Table 1 can be simply viewed as an asperity peak of 3.92 μm that was compressed down to 0.19 μm, corresponding to a true compressing strain *ε* = ln(3.92/0.19) = 3.0. The staining time, however, was rather complicated, since the sample rotated with *V*_1_ = 68 rpm moved at *V*_2_ = 10 mm/min, vibrated at 20 kHz, and was processed by four passes. Each turn, the peak was flatted by the roller one time in a duration that can be estimated according to the peak range (*l_a_*), the perimeter of the inner circle (*l_p_*), the rotation velocity (*V*_1_): *t* = *l_a_*/(*l_p_* × *V*_1_) = 103.64/(251,200 × 68 ÷ 60) = 3.6 × 10^−4^ s, assuming that the peak range was equal to the peak distance (*Rs*). From the start to the end of processing, the roller moved along the pipe axis at *V*_2_ for 2 mm, for a duration of 2/(10 ÷ 60) = 12 s, during which the sample rotated 12 × 68/60 = 13.6 turns. This means that the asperity peak was flatted for 13.6 × 3.6 × 10^−4^ = 4.9 × 10^−3^ s after one pass processing, and the total straining time after 4-pass processing was *t_tot_* = 2.0 × 10^−2^ s. As a result, the average local bulk strain rate $\dot{\varepsilon }$ can be 150 s^−1^. The average flattening velocity *v_fs_* can be estimated to be Δ*Ra*/(*t_tot_*) = 186.5 μm/s. When *S_m_* = *Rs* = 103.64 μm, the non-dimensional *E* can be 41.7. For processing with normal surface rolling, the *Ra* changed from 3.92 to 1.49 μm, and *Rs* remained 103.64 μm. Under such condition, $\dot{\varepsilon }$ = ln(3.92/1.49)/(2.0 × 10^−2^) = 48.2 s^−1^, *v_fs_* = 2.43/(2.0 × 10^−2^) = 121.5 μm/s, *E* = 20.6, and *t_tot_* remained 2.0 × 10^−2^ s. The application of ultra-sonic vibration raised the non-dimensional strain rate *E* by a factor of 2. It should be noted that the asperity peak was totally flattened by 2 × 10^−2^ s × 20,000 = 400 times, and the flattening decreased and eventually reached zero as the flattening became increasingly more difficult. This implies that the starting flattening had the strain rate that was much higher than the present estimation, even though the low bound estimation also underpinned the ultra-sonic effect in promoting the flattening process through high strain rate-reduced effective asperity hardness. During USRT, sample surface experienced rolling pressure supplemented with extra cyclical pressure due to ultrasonic vibration. The instantaneous loading onto the asperity peak was expected higher than that of the normal surface rolling. Moreover, under cyclical pressure with cycles of 20,000 per second, the asperity peak was punched hundreds of times in 20 milliseconds and could easily deform to fracture, resembling the compression fatigue behaviors. High frequency loading also induced more significant local temperature rising that could soften the asperity peak as well. During dynamic plastic deformation at strain rate of 10^2^–10^3^ s^−1^, the bulk temperature of pure Ni was increased by 40–73 K [19]. On the other hand, for AISI 304 stainless steel subjected to surface shearing with strain rate of 10^3^–10^4^ s^−1^, surface temperature rising can be tens to a hundred degrees [14]. The temperature rising for local asperity peak is expected to be more pronounced. Consequently, asperity flattening by USRT is more effective.

**Figure 13:**
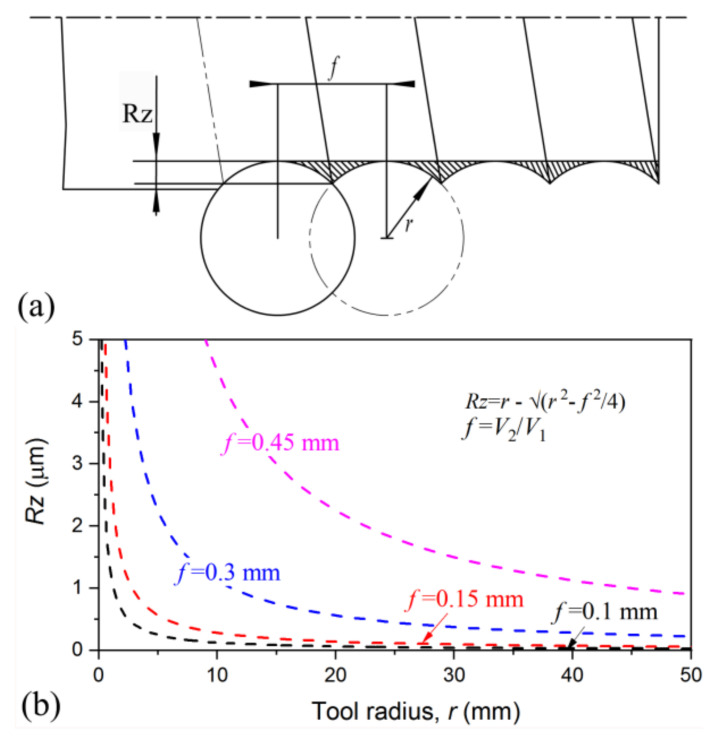
Schematic illustration of the surface roughening during processing by tool with radius *r* (**a**) and the variation of roughness as a function of *r* for different feed rates (**b**).

Moreover, the advantage of processing by roller with line-contact was highlighted. As shown in Figure 13a, the rotated smooth surface generated roughness after the processing by a smooth tool with a tip radius *r*. The distance between the remaining peak and valley *R_Z_* can be roughly estimated, with $Rz=r-\sqrt{r^{2}-{\left(\frac{f}{2}\right)}^{2}}$, where *f* is the feed rate. For the present case, *f* = *V*_2_/*V*_1_ = 10/68 = 0.15 μm. As seen in Figure 13b, the surface roughness increased with an increase of feed rate, implying that fast rotation and slow moving benefits surface quality. Given feed rate *f*, surface roughness decreased sharply with an increase of tool radius, and for *r* = ∞, namely, line-contact, *R_Z_* theoretically approached zero asymptotically.

USRT showed a unique advantage in generating mirror surface with effective structural modification. Firstly, USRT is a debris-free processing that flattens surface through deformation of the asperity peak. However, compared with the traditional surface rolling, USRT flattens surface with very smaller stress that is unable to bring effective smoothening by traditional surface rolling without ultra-sonic vibration. As demonstrated by the roughness measured in Table 1, the traditional surface rolling produced *Ra* = 1.49 μm that was 7.8 times larger than that for USRT. This advantage enabled USRT to produce less surface damage than that for traditional surface rolling, which needs large stress to flatten the surface. Moreover, USRT can be applied for soft materials or thin-wall pipes that are unable to be processed by the traditional surface rolling. Secondly, USRT produces gradient hardened surface of 600 μm thick with a maximum hardening by a factor of 2–3. This is very superior to that for most mirror-surface processing techniques such as surface polishing or grinding, which commonly generate insignificant hardening. The effective hardening together with the good surface quality may find direct applications for products demanding high hardness against surface wearing. According to Table 1, surface quality produced by USRT was very close to that of the machining roller, which indicates that the surface quality treated by USRT may be further improved with the improvement of the machining roller. This optimize behavior cannot be realized by most traditional processing techniques. Thirdly, USRT can be used as a final processing to enhance the service behavior. USRT can install on the machine tool, turning machine, grinding machine, etc. to perform final surface modification without disassembling the workpiece. This advantage raises the processing efficiency, and the size of workpiece can be easily guaranteed since secondary alignment and processing are unnecessary. Finally, USRT may benefit to the surface chemical heat treatment such as nitriding, chromizing, and carburizating. Owing to the structural nanostructuring, a high density of boundaries and dislocations were formed to act as the fast diffusion paths, which leads to atomic diffusion at relatively lower temperature and shorter time [23]. As a result, USRT may find wide applications in the precious machining fields of basic parts such as bearings, gears, pistons, and hydraulic components. These parts are widely used in aerospace, automobile, petrochemical, high-speed rail, and other industries. Their surface quality is the basis for the survival and development of the equipment manufacturing industry, and their level directly determines the quality, life, and reliability of major equipment and host products.

### 4.2. Structural Evolution Process

Plastic deformation decreases with the depth and the microstructures along the depth, thus record the process of structural evolution toward a finer scale. In a length scale covering millimeters to nanometers, the deformation of AISI 304 stainless experiences the deformation mechanism of typical low stacking fault energy (SFE) metals, following that of high-SFE metals. Specifically, the structure evolution involves four stages: (i) generation of planar dislocation arrays and deformation twins, (ii) formation of twin bundles, (iii) initiation and development of SBs, and (iv) formation of ultrafine- and nano-laminated structure.

The first deformation stage is well documented in previous publications [14,15,16,20], characterizing the low strain deformation of low-SFE metals. The formation of planner dislocation arrays has the cause related to the low-SFE-suppressed dislocation cross-slip that is a prerequisite for the formation of dislocation cells for high-SFE metals [24]. Dislocations were restricted on their respective {111} slip planes, exhibiting a planar configuration. For USRT AISI 304 stainless steel, planar dislocation arrays occurred at a far deep layer (>450 μm), where applied loading was so low that dislocation was activated to accommodate the plastic strains. As the stress at smaller depth reached the critical resolved shear stress for twinning, deformation twins were activated to realize the first subdivision by twin boundaries, i.e., special high-angle boundaries with axis/angle of <111>/60°. Multiple twins were activated when the resolved shear stress reached the critical point for other twinning systems, and the twin boundary subdivision became more significant. Such a grain-subdivision process via twin boundaries can operate down to the nano-scale when the sample surface was bombarded multidireactionally by flying shots with high speed, so that more and more twins were continuously formed [15]. USRT deforms the surface by rolling supplemented with cyclical compressing, of which the loading direction remains the same during the cyclically changed loading amplitude. It is expected that the twining systems that were stimulated by the first cycle will be activated repeatedly before grains change their orientations to activate other twin systems. This is supported by Figure 3, showing the less evidenced change in the grain’s geometrical shape, which was very different from the substantially sheared original grains in PISG processed AISI 304 stainless steel [14,20]. As a result, the active sources operated repeatedly to generate new twins, forming twin bundles with high density of twin boundaries to subdivide the grains. This phenomenon is very prevalent, as demonstrated by Figure 7, showing that twin bundles occurred frequently, whereas the adjacent regions did not have significant twin multiplication. The mechanism for formation of new twins is at present not clear, but it might follow the partial dislocation slip mechanisms in coarse-grained fcc metals, including pole [25], prismatic glide [26], and faulted dipole [27], which require the partial dislocation slip continuously on consecutive {111} slip planes. Moreover, boundary-related twinned mechanism should also be activated, such as coincidental overlapping of wide stacking fault ribbons from grain boundaries [28], grain boundary splitting and migration [29], and random activation of partials from grain boundaries [30]. The continuous formation of new twins is responsible for the evolution of coarse-twin region to fine-twin region in the depth range of 450–50 μm (Figure 3).

**Figure 14:**
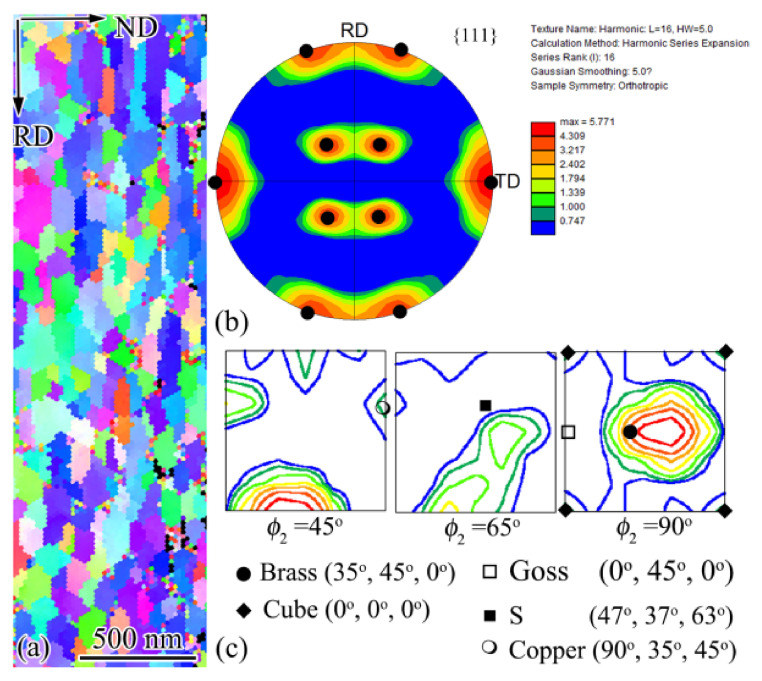
TKD analysis of the ultrafine laminated structure on the surface layer in USRT AISI 304 stainless steel sample: IPF image (**a**); {111} pole figure (**b**); and ODF plots for ϕ_2_ = 45°, 65°, and 90°, respectively (**c**). Typical rolling texture components, i.e., Brass, Goss, Cube, S, and Copper were inserted for comparison. Note that UFL shows typical Brass-type rolling texture.

After a high density of single- or multiple-twins were formed within the coarse grains, deformation became localized and the plastic flow is concentrated in narrow regions, forming SBs that are composed of layered structures [31]. The layered structure is parallel with the shear direction that is commonly aligned with one of the maximum shear plane; for rolling, it is ±35° to the rolling direction. However, as shown in Figure 3, SBs at larger depth were inclined 20° to the rolling direction, while at smaller depth, they were roughly parallel with the surface, and both deviated from the maximum shear plane. This implies the deformation-induced change in crystal orientation that plays a decisive role in governing the deformation microstructure [32]. The nucleation and propagation of SBs within TS were expected to be identical to those for AISI 304 stainless subjected to the PISG at room temperature, including the destroying TS via local shearing-induced interaction between dislocation slip and twins; the formation, reorientation, and the detwinning of deformation twins within SBs; and finally the formation of dislocation boundaries [20]. The formation of dislocation boundaries characterizes the structural evolution for typical high-SFE metals [4,33,34]. The continuous formation of SBs gradually replaces the TS by layered structures that were characterized by UFL at a larger depth and NL at a smaller depth. The SBs generated in the present USRT sample resembled those for traditional cold rolled ones, being typical Brass-type [35]. As demonstrated by the inverse pole figure (IPF) (Figure 14a) obtained using transmission Kikuchi diffraction (TKD) that has high space and angular resolution, UFL were found to be parallel with the rolling direction (RD), giving an {111} pole figure (Figure 14b) and orientation distribution function (ODF) image (Figure 14c) that showed typical Brass-type texture.

## 5. Conclusions

An AISI 304 stainless steel pipe inner surface was processed by 4-pass USRT at room temperature. The microstructure and hardening along the depth were systematically characterized, and the surface quality was evaluated. The following conclusions were reached:

(1) USRT generated hardened layer of 600 μm thick and GNS of 20–30 μm thick. The surface microstructure was refined down to 42.3 nm, showing a hardness of 450 HV that was more than two times higher than that of the starting counterparts (190 HV).
(2) USRT greatly enhanced the surface quality. Compared to the starting turning samples, *Ra* decreased by a factor of 20 from 3.92 to 0.19 μm, which was 7.8 times lower than that (1.49 μm) for rolling without ultrasonic vibration. The mechanism was proposed to be an asperity peak flattening process, of which the non-dimensional asperity hardness was reduced due to the ultrasonic vibration-induced high non-dimensional strain rate.
(3) Structural evolution from millimeter- to nanometer-scale was established. There were four stages: (i) generation of planar dislocation arrays and deformation twins, (ii) formation of twin bundles, (iii) initiation and development of SBs, and (iv) formation of ultrafine- and nano-laminated structure.

## Figures and Tables

**Table 1:** Surface roughness for the AISI 304 stainless steel pipe inner surface before and after the RT and USRT processing.

	*Ra* (µm)	*Rz* (µm)	*Rz (JIS)* (µm)	*Rs* (µm)	*Rp* (µm)
Turning	3.92	19.14	15.78	103.64	9.08
After RT	1.49	8.11	6.76	88.18	1.86
After USRT	0.19	1.43	0.69	75.56	0.39
Turning/RT	2.6	2.4	2.3	1.2	4.9
Turning/USRT	20.6	13.4	22.9	1.4	23.3
RT/USRT	7.8	5.7	9.8	1.2	4.8

## Data Availability

Data can be available upon request from the authors.
